# Linking Physical Activity to Personal Values: Feasibility and Acceptability Randomized Pilot of a Behavioral Intervention for Older Adults with Osteoarthritis Pain

**DOI:** 10.21203/rs.3.rs-1182374/v1

**Published:** 2022-01-03

**Authors:** Jennifer C Plumb Vilardaga, Sarah Kelleher, Allison Diachina, Jennie Riley, Tamara Somers

**Affiliations:** Duke University Health System; Duke University School of Medicine; Duke University School of Medicine; Duke University School of Medicine; Duke University School of Medicine

**Keywords:** Osteoarthritis, Pain, Physical Activity, Values

## Abstract

**Background:**

Osteoarthritis (OA) pain is common and leads to functional impairment for many older adults. Physical activity can improve OA outcomes for older adults, but few are appropriately active. Behavioral interventions can reduce barriers to physical activity. We developed and tested a brief, novel behavioral intervention for older adults combining values to enhance motivation and strategic activity pacing to improve arthritis-related pain and functioning and increase physical activity.

**Methods:**

A randomized feasibility and acceptability pilot trial compared Engage-PA to treatment as usual plus fitness tracker (TAU+) in N=40 adults age 65+ with OA pain in the knee or hip. Engage-PA involved two 60-minute telephone sessions. All participants wore a fitness tracker to collect daily steps throughout the study and completed baseline and post-treatment assessments of secondary outcomes (arthritis-related pain and physical functioning, physical activity, psychological distress, psychological flexibility, and value-guided action). The impact of COVID-19 on general wellbeing and physical activity was also assessed. Descriptive statistics were conducted for feasibility and acceptability outcomes. Indicators of improvement in secondary outcomes were examined via change scores from baseline to post-treatment and performing independent samples t-tests to assess for between-group differences.

**Results:**

Feasibility was high; 100% accrual, low (5%) attrition, and 100% completion of study sessions. Acceptability was high, with 89% finding the intervention “mostly” or “very” helpful. Engage-PA participants demonstrated improvements in arthritis pain severity (*M*_diff_=1.68, *p*<.05), arthritis-related physical functioning (*M*_diff_=.875, *p*=.056), and self-reported activity (*M*_diff_=.875, *p*<.05) from baseline to post-treatment as compared to TAU+. Sixty-three percent of participants provided useable objective daily steps data. Other secondary outcome patterns were not interpretable in this small sample. COVID-19 added additional burden to participants, such that 50% were exercising less, 68% were more sedentary, and 72% lost access to spaces and social support to be active.

**Conclusions:**

Engage-PA is a promising brief, novel behavioral intervention that has potential to support older adults in improving arthritis-related pain and functioning and increasing physical activity. The feasibility and acceptability of the intervention is particularly notable as most participants reported COVID-19 added more barriers to physical activity, and Engage-PA may be appealing in future studies.

**Trial Registration:**

clinicaltrials.gov, NCT04490395, registered 7/29/2020, https://clinicaltrials.gov/ct2/show/NCT04490395.

## Background

Osteoarthritis (OA) is one of the most common age-related problems facing older adults,(1) and is often a progressive, degenerative condition.(2) Treatments such as surgery and medications are available, but older adults are at particular risk for more complications and poorer functional outcomes from joint replacement surgery(3) and the rate of complications and mortality from problematic opioid therapy (e.g., chronic, high dose) is increasing.(4) OA can lead to persistent pain and declining physical functioning for many older adults.

Physical activity is a low-cost, low-burden approach commonly recommended for older adults with OA as treatment for pain, prevention of disability, and for improving outcomes following joint replacement.(5–7) Importantly, physical activity can be conducted safely for even vulnerable older adults.(8) Physical activity is critical for preserving physical functioning in older adults; lack of exercise contributes to decreased mobility and persistent pain, reducing the likelihood of independent living and exacerbating other common health conditions.(9)

Yet few older adults with OA engage in recommended levels of physical activity, with common barriers including persistent pain and distress.(10) Behavioral interventions can overcome these barriers by teaching skills to cope with pain and distress to allow for regular engagement in physical activity.(11, 12) There is a need for the development and implementation of behavioral approaches that will address these barriers to improve arthritis-related pain and functioning and increase physical activity in this population.

Increasing physical activity, like any health behavior change, involves both motivation for change and specific skills to initiate and sustain the behavior change. Identifying and setting goals based on *personal values,* or deeply held, personally-chosen statements of meaning and purpose, is one potentially crucial facet of motivation and commitment for health behavior change.(13–15) Acceptance and Commitment Therapy (ACT(16)) is a behavioral approach that has an explicit, theory-derived focus on personal values. (17, 18) ACT is unique in that its mechanism of change is psychological flexibility, defined as persistence towards one’s stated values even when psychological barriers (i.e., emotional distress, negative thoughts, pain) and other external challenges (i.e., time) arise.(19) There is a growing body of work using these methods, even in brief formats, demonstrating improvements in health behaviors including smoking,(20) weight loss(21), and physical activity(22, 23). Values-based approaches have promise for adults with pain,(24) though one study using these methods with adult veterans (many of whom are older adults) with persistent pain only demonstrated benefits on psychological variables and not in physical activity levels.(23) These researchers recommend that values-based behavioral interventions should increase their focus on physical activity promotion.(23)

*Activity pacing* is one critical behavioral skill for increasing physical activity that may assist older adults with OA in engaging in physical activity. The activity-rest cycle is a form of activity pacing that is an important component of behavioral symptom management programs (e.g., Pain Coping Skills Training [PCST(25)]) for patients with disease-related pain (e.g., cancer(26–31) and arthritis(12, 32, 33)). The activity-rest cycle is rooted in social cognitive theory, (34) with a particular focus on increasing self-efficacy to engage in a particular pain management task. This skill encourages sustaining smaller amounts of activity more consistently over time, and slowly building up levels of activity and reducing resting periods (vs. overdoing that leads to increased pain and avoidance of activity). Combining personal values and activity pacing has a promising synergy that may be very well suited to helping older adults with OA pain to improve arthritis-related pain and functioning and increase physical activity.

Our team developed such a novel, combined protocol (i.e., Engage) to manage pain, fatigue and distress for those with advanced cancer, many of whom were older adults, with promising outcomes.(35, 36) Extending this work to patients with OA-related pain, we adapted the protocol (i.e., Engage-PA) to meet the needs of older adults and integrated values and activity pacing for the express purpose of increasing daily steps. Engage-PA also incorporated wearable fitness trackers to allow participants to self-monitor their daily steps.

The primary aim of the current study was to examine the feasibility and acceptability of delivering Engage-PA to older adults with OA pain. Our hypotheses were that Engage-PA would be feasible for future larger-scale studies if 1) we achieved at least 75% accrual of our planned N of 40, attrition was less than 20%, and at least 75% of participants completed both study sessions, and 2) acceptability of the intervention was shown with at least 80% reporting the intervention mostly or very helpful. Our secondary aim was to examine changes in arthritis-related pain and functioning, physical activity, psychological distress, psychological flexibility, and valued-living before and after patients engaged in the intervention. Given the timing of the study, we also included a measure of COVID-19 impact to assess patients’ experience with exercise routines, sedentary behaviors, health and illness, or other losses/instability due to the pandemic.

## Methods

### Participants & Procedures

The current study was a randomized pilot feasibility and acceptability trial of older adults (N=40) with a diagnosis of OA in the knee or hip comparing Engage-PA to treatment-as-usual plus fitness tracker control. In line with CONSORT guidelines,(37) as a pilot feasibility study, a formal power calculation for sample size was not required. Sample size was selected based on previous successful pilot work conducted by our team with similar feasibility pilot goals and planned secondary outcome analyses. (35,36) Participants were recruited from September 2020 through August 2021. Duke University Institutional Review Board provided ethical approval (Pro00105573) and the trial was registered on www.clinicaltrials.gov (NCT04490395).

Potential participants were identified by electronic medical record review based on inclusion and exclusion criteria. Potential participants were contacted by trained study staff via telephone and/or email summarizing study information and inviting participants to answer screening questions either online or over the phone. Inclusion criteria included (1) adults aged 65 or older, (2) diagnosis of OA in the knee and/or hip, (3) English speaking, (4) ability to participate in telephone sessions, (5) ability to ambulate even if assisted by a cane or walker, (6) rating worst pain and pain interference within the last week as a 3 or greater out of 10. Exclusion criteria included (1) planned surgery during the study duration that would limit mobility for more than 3 weeks, (2) major surgery requiring limited mobility within the past 3 months, (3) myocardial infarction within the past 3 months, (4) fall(s) within the past 3 months that led to immediate medical treatment, (5) current enrollment in cardiac rehabilitation, (6) presence of a serious psychiatric condition, (7) reported or suspected moderate cognitive impairment, (8) indication by a medical provider that exercise should only be medically supervised, and (9) presence of other unmanaged medical condition (e.g., hypertension, diabetes, asthma, neurodegenerative condition) that might lead to unsafe participation as outlined in the Physical Activity Readiness Questionnaire Plus (PARQ-2020;(38) an evidence-based measure for patient-determined safety for engaging in physical activity) subsequently verified by electronic medical record review and/or via communication with patients’ treating medical team.

Interested and eligible participants were invited to engage in consent procedures. Consent was conducted online (via Research Electronic Data Capture [REDCap], a HIPAA-compliant secure online data collection and management tool), or via telephone or mailed paper packets. After consent, participants completed baseline and post-treatment assessments online via REDCap or via telephone or mailed paper packets. All participants received a personal fitness tracker device which provided participants an objective measure of their daily steps. Study staff managing recruitment, assessments, intervention sessions, and fidelity checks were not blinded to condition. Assessment data was entered into REDcap directly by participants wherever possible. Following completion of baseline assessment, participants were randomized to either active treatment (Engage-PA) condition or treatment-as-usual with fitness tracker (TAU+) control group. Randomization was conducted using Randomization Windows Version 5.0 with equal allocation to groups. Study staff who had no contact with study participants conducted randomization; condition assignment was populated in REDcap automatically.

**Figure 1:**
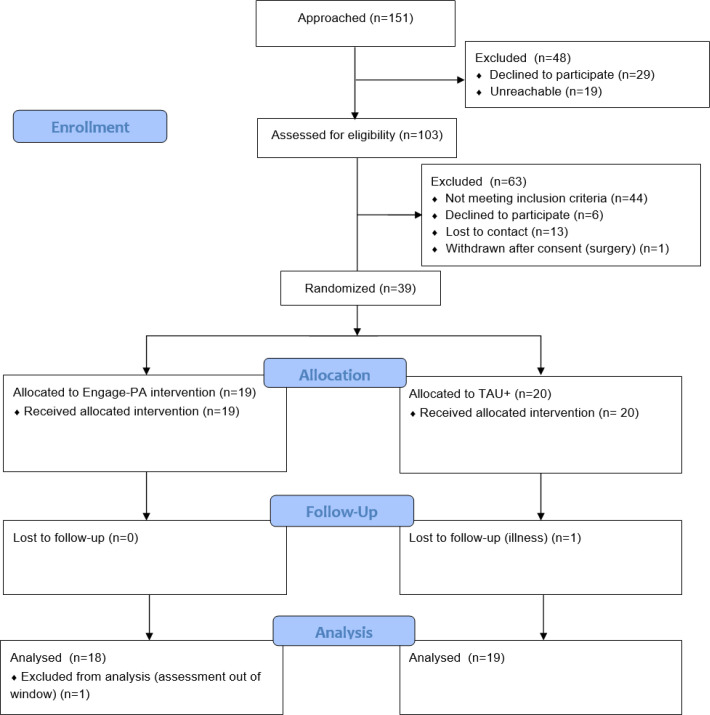
CONSORT flow diagram

Participants in the intervention group received a study workbook to guide their two 45-minute telephone-delivered treatment sessions, spaced approximately 2 weeks apart. To set up later implementation efforts, the intervention sessions were delivered by a master’s level study therapist, guided by a written treatment manual, audio recorded, and checked for fidelity by a senior researcher. Participants in both groups reported on their daily steps as collected by the personal fitness tracker. All participants were compensated $30 for each completed assessment. Participation in this study lasted approximately 6 weeks. Figure 1 CONSORT diagram provides additional details about participant flow through the study.

### Intervention

In Session 1, participants first discussed the fitness tracker and their baseline level of daily steps. The study therapist then explored the participant’s experience with OA pain and physical activity, and introduced personal values and activity-rest cycle skills. Personal values were defined as guiding principles that are likely to bring meaning and purpose. Participants were asked to identify two personal values: one related to physical activity and one related to another life domain (e.g., relationships). Personal values were linked to goal setting to allow for more purposeful action, which may increase meaning and satisfaction. Participants were then asked to reflect on their experience with over-doing activity (often resulting in increased pain) or under-doing activity (often resulting in deconditioning), and were subsequently introduced to the activity-rest cycle. The study therapist worked with each participant to identify timed activity bouts and timed strategic rest breaks so as not to exacerbate pain.Participants used the activity-rest cycle to generate specific, timely, and measurable goals related to physical activity (e.g., walk for 10 minutes) and another value-guided action (e.g., observe a grandchild’s sports event).

In Session 2, conducted two weeks after Session 1, participants reflected on their successes and barriers in doing their specific goals for physical activity and other value-guided activity. The activity rest cycle was troubleshooted in this session, with challenges, successes, and alternative strategies presented. Participants’ specific values were again discussed, and time was spent considering ways to refine values and actions to promote success (or increase physical activity). Continuing to engage in valued activity even if pain or psychological distress increased was highlighted, and the activity-rest cycle was presented as one way to do this.

### Primary Outcome Measures

#### Feasibility and Treatment Acceptability.

Feasibility was measured by participant enrollment (accrual), attrition, and session attendance. Acceptability was measured by The Client Satisfaction Questionnaire (CSQ)(39,40)post-treatment. The CSQ is commonly used in treatment acceptability studies and includes items such as, “How satisfied are you with the amount of help you received?” and “To what degree did the program teach you skills that are helping you to better manage your symptoms?” rated on a Likert scale.

### Secondary Outcome Measures

#### Arthritis-Related Pain and Functioning.

The Arthritis Impact Measurement Scale (AIMS)(41) was used to assess arthritis pain and functioning. Respondents answer questions on a 5-point Likert scale where higher scores indicate greater arthritis-related impact on functioning and health. The Symptom subscale includes items of pain frequency, pain severity, sleep interference from pain, and morning stiffness. The Physical Functioning subscale includes items of arthritis-related mobility, walking and bending, self-care activities of daily living, and household tasks. The AIMS is commonly used to assess functioning related to OA(41).

#### Physical Activity (objective and self-report).

Physical activity was measured via both self-report and objective daily step measures.

##### Rapid assessment of physical activity (RAPA).

(42) The RAPA is a 9-item self-report measure where respondents answer a series of Yes/No questions about their engagement in a range of aerobic (e.g., light, moderate, vigorous) and non-aerobic (e.g., yoga/flexibility, strength-training) activities, as well as frequency of their engagement in these activities. Higher scores indicate more activity. The RAPA was designed and validated for use with older adults.(42)

##### Daily steps.

Participants used a wearable watch-style consumer-grade fitness tracker, a Garmin VivoFit 4.0 ©, upon waking and until bedtime daily throughout the study. Daily step counts were compiled for seven consecutive days at baseline and seven consecutive days post-treatment. Participants were mailed a daily step log, which they used to write down the daily step reading at bedtime from the Garmin device. Participants communicated with study staff via phone or email to report daily step counts.

#### Psychological Distress.

The AIMS(41) Affect subscale was used to measure overall psychological distress related to anxiety, tension, and depressed mood. As in the other AIMS subscales, respondents answer questions on a 5-point Likert scale where higher scores indicate greater arthritis-related impact on functioning and health.

#### Psychological Flexibility.

The Acceptance and Action Questionnaire-II (AAQ-II)(43) was used to assess overall psychological flexibility, or the ability to accept difficult psychological experiences as one lives in line with important life goals. In the AAQ-II, participants report on their emotional experience (e.g., “I worry about not being able to control my worries and feelings”) on a scale from 1 = “Never true” to 7 = “Always true”. The AAQ-II has commonly been used as a process of change measure in ACT-based studies and has been related to changes in symptom severity.(44,45)

#### Valued-Living.

The Bulls-Eye Values Survey(46,47) was used to assess how consistently patients had been living in line with their chosen values. First, patients write about their personal values for living life with meaning and purpose across domains of relationships, education/work/community, leisure, and health/well-being, and then rate how successful they have been living in line with these values in the last month. A dartboard image is provided and responses ranged from 1 = “a perfect bulls-eye, and great success living in line with a value” to 8 = “very far away from living in line with a value”. This measure has demonstrated utility for pain populations(36) and other behavioral medicine populations.(46,47)

### COVID-19 Impact Measure

The impact of the COVID-19 pandemic was assessed at baseline for all participants using the COVID-19 Impact Scale (CIS)(48) which was developed and distributed as part of the National Institutes of Health Office of Behavioral and Social Sciences Research toolkit. The CIS was used to assess overall COVID-19 impact on routines, health, mental health, personal or family member COVID-19 illness/death, and areas of life insecurity (e.g., finances). Additional items were developed for this study, which assessed changes since the pandemic in amount of exercise, amount of time spent in sedentary activity, and other ways in which the pandemic affected exercising (e.g., access to fitness centers, social support for exercising).

### Changes to Assessments and Measurement

Two changes to study assessments and measures occurred after the pilot trial began. First, ActiGraph© accelerometers were initially used as an additional objective measure of physical activity (daily steps, time spent in moderate, vigorous or light/sendentary activity), but due to pandemic related challenges with remote procedures, two-thirds of the participants did not wear accelerometers. Additionally, a third assessment (1 month follow up) with both self-reported and objectively-measured (i.e., daily step count) secondary outcome measures was discontinued after the pilot trial began.

### Analytic Strategy

Primary feasibility and acceptability outcomes were examined using descriptive statistics. For secondary outcomes, paired sample t-tests were calculated to examine between group changes from pre- to post-intervention in arthritis-related pain and functioning, psychological wellbeing, valued living, and daily steps. Secondary outcome analyses were conducted to examine possible indicators of improvement, and given the small sample size should be interpreted with caution.(49)

## Results

In Session 1, participants first discussed the fitness tracker and their baseline level of daily steps. The study therapist then explored the participant’s experience with OA pain and physical activity, and introduced personal values and activity-rest cycle skills. Personal values were defined as guiding principles that are likely to bring meaning and purpose. Participants were asked to identify two personal values: one related to physical activity and one related to another life domain (e.g., relationships). Personal values were linked to goal setting to allow for more purposeful action, which may increase meaning and satisfaction. Participants were then asked to reflect on their experience with over-doing activity (often resulting in increased pain) or under-doing activity (often resulting in deconditioning), and were subsequently introduced to the activity-rest cycle. The study therapist worked with each participant to identify timed activity bouts and timed strategic rest breaks so as not to exacerbate pain. Participants used the activity-rest cycle to generate specific, timely, and measurable goals related to physical activity (e.g., walk for 10 minutes) and another value-guided action (e.g., observe a grandchild’s sports event).

In Session 2, conducted two weeks after Session 1, participants reflected on their successes and barriers in doing their specific goals for physical activity and other value-guided activity. The activity rest cycle was troubleshooted in this session, with challenges, successes, and alternative strategies presented. Participants’ specific values were again discussed, and time was spent considering ways to refine values and actions to promote success (or increase physical activity). Continuing to engage in valued activity even if pain or psychological distress increased was highlighted, and the activity-rest cycle was presented as one way to do this.

### Participant Characteristics

Demographic information was collected from 39 of the 40 participants (one participant withdrew prior to baseline) and are indicated in Table 1. Participants were aged 65 and older (*M*=71.77, range 65–90), mostly female (84.6%), with 62% percent self-identifying as white and 33% self-identifying as Black or African-American. Thirty-seven percent reported an annual household income of less than $60,000/year. The majority of participants had OA in the knee (64%), with 28% reporting OA in both knee and hip. The most common medical co-morbidities reported were hypertension (54%), depression (31%), anxiety (23%) and diabetes (21%).

#### COVID-19 Impact.

Forty-one percent of participants reported that COVID-19 impacted one or more life areas in a severe way (e.g., finances, social life, mental health, healthcare access, personal or family member COVID-illness). In comparison to before the pandemic, 72% reported losing access to locations (e.g., gyms, pools, large indoor walking spaces) or social support for exercising, 68% reported being more sedentary, and 52% of individuals reported exercising less than before the pandemic. Additional participant characteristics are outlined in Table 2.

### Feasibility and Acceptability Results

Feasibility was demonstrated across several metrics. First, all 40 participants (100% accrual) were enrolled within the study timeframe. Attrition was low (5%), with only 2 withdrawals; one participant before baseline assessment or randomization (due to surgery) and one between baseline and post-treatment assessment (due to illness). All participants assigned to the Engage-PA intervention condition completed both sessions (100% treatment completion).

Acceptability was high, with 89% of Engage-PA participants finding the intervention “mostly” or “very” helpful. Examples of helpful elements reported were “encouragement,” “setting regular walking goals,” and “clarifying values...to live my best life”. Areas for improvement included suggestions for greater clarity on topics discussed, additional topics to include, and concerns about device use.

### Secondary Outcomes

Engage-PA participants demonstrated improvements in arthritis pain (*M*_diff_=1.68, *p*<.05) from baseline to post-treatment as compared to TAU+. A trend toward improvement in arthritis-related physical functioning (*M*_diff_=.875, *p*=.056) was also found for the Engage-PA group. Engage-PA participants also demonstrated improvements in self-reported physical activity (*M*_diff_=.875, *p*<.05) from baseline to post-treatment as compared to TAU+ participants. Sixty-three percent of participants provided objective measurements of daily steps data, despite device complications and challenges in syncing devices. Descriptive data on other secondary outcomes was not interpretable in this small sample. Results are detailed in Table 3.

## Discussion

This is the first study to examine a novel, brief, combined values and activity-pacing intervention for improving arthritis-related pain and functioning and increasing physical activity in a sample of older adults with osteoarthritis pain. Engage-PA demonstrated high levels of feasibility and acceptability, and there were indications of improvement in secondary outcomes of arthritis-pain and functioning, as well as self-reported physical activity.

It is notable that Engage-PA was feasible even though the study was conducted entirely during the COVID-19 pandemic, especially because most participants reported that the pandemic had a negative impact on general wellbeing and physical activity. Most had less access to locations to exercise or social support for exercising and were more sedentary due to the pandemic, and 41% reported severe impact from COVID on their health, finances, or other life areas. This indicates that they had more barriers to overcome in order to engage in physical activity than others with OA pain in studies conducted prior to the COVID-19 pandemic. Yet despite the significant pandemic-related problems with exercising, participants in Engage-PA were recruited, enrolled, retained, and completed sessions at high rates comparable to studies conducted under more ideal circumstances. This may be related to participant feedback that Engage-PA provided not only important motivation and pacing skills for increasing physical activity, but also additional support, problem-solving, and accountability for meeting physical activity goals. Given that older adults with OA pain have long reported significant challenges with initiating and maintaining physical activity, these findings during the COVID-19 pandemic suggest Engage-PA would be feasible and acceptable in the future as well.

Engage-PA shows particular promise for larger scale implementation. The study was successful in recruiting participants from diverse backgrounds in line with demographics of patients served in the treating clinics, although most enrolled participants were female. Future studies should seek to recruit from a wider array of participants, particularly those from historically marginalized backgrounds or with less access to care. It is a brief protocol, delivered by master’s level behavioral interventionist, with many formalized elements (i.e., manual, workbook, personal fitness tracker); features that may be appealing for primary care clinic dissemination. Finally, it was delivered with completely remote procedures, including telephone and online recruitment, telephone and online enrollment, telephone session delivery, and remote assessment completion (online, telephone-collected or mailed paper packets). There were some challenges related to fitness-tracker device use linked to difficulties with syncing devices for remote data collection. However, participants largely enjoyed having personalized daily steps feedback. With some small changes to study procedures, the personal wearable fitness-tracker may provide a more reliable, robust measure of daily steps in future studies.

## Conclusions

Engage-PA is a novel, brief behavioral intervention for older adults with OA pain that demonstrates high feasibility and acceptability even when patients present with considerable barriers to establishing physical activity routines (e.g., during the COVID-19 pandemic). Since feasibility and acceptability criteria were met but some features of the protocol need refinement, next steps should include refinement of protocol based on participant feedback followed by larger effectiveness studies in real-world clinical care settings. If found effective in future studies, Engage-PA may provide important opportunities for older adults to reduce arthritis-related pain, increase physical activity and improve day to day function.

## Supplementary Material

Supplement 1

## Figures and Tables

**Table 1.** Demographic Characteristics (N=39).

	N (%)	*M*(*SD*)

**Age** (years)		71.77 (5.198)

**Gender**		
*Male*	6 (15.4%)	
*Female*	33 (84.6%)	
**Race**		

*Caucasian/White*	24 (61.5%)	
*Black or African-American*	13 (33.3%)	
*2 or more races*	2 (5.1%)	

**Ethnicity**		

*Non-Hispanic*	37 (94.9%)	
*Hispanic or Latino*	2 (5.1%)	

**Education**		

*Less than High School Diploma*	1 (2.6%)	

*High School Diploma*	2 (5.1%)	

*Some College*	7 (17.9%)	

*Bachelor’s Degree*	8 (20.5%)	

*Graduate Degree*	21 (53.8%)	

**Income**		

*$10,000 to $19,999*	5 (12.8%)	

*$20,000 to $39,999*	6 (15.4%)	

*$40,000 to $59,999*	2 (5.1%)	

*$60,000 to $100,000*	9 (23.1%)	

*More than $100,000*	13 (33.3%)	

Note. *M* = mean; *SD* = standard deviation.

**Table 2.** Participant medical characteristics and COVID-19 impact (N=39).

**Medical Characteristics**	**N (%)**
OA location	
*Knee*	25 (64%)
*Hip*	3 (8%)
*Both*	11 (28%)
Hypertension	21 (54%)
Heart Disease	5 (13%)
Rheumatoid Arthritis	4 (10%)
Diabetes	8 (21%)
Sciatica	9 (23%)
Emphysema, asthma, or COPD	3 (8%)
Depression	12 (31%)
Anxiety	9 (23%)
Stroke or brain bleed	1 (3%)
Cancer (past or current)	6 (15%)
**COVID-19 Impact**	**N (%)**
Reduced access, support for exercise	28 (72%)
Exercising less than prepandemic	20 (52%)
More sedentary than prepandemic	26 (68%)
Same/less sedentary than prepandemic	13 (33%)
Severe COVID impact in 1+ life area	16 (41%)

Note. OA=osteoarthritis; COPD=chronic obstructive pulmonary disease. COVID-19=coronavirus disease.

**Table 3.** Secondary outcome results.

	*M*_diff_	*p*	CI (95%) Lower, Upper
Arthritis Pain (AIMS symptom subscale)	1.68	.044	−0.26, 3.62
Physical Functioning (AIMS PF subscale)	0.59	.056	−0.15, 1.33
Physical Activity (RAPA)	−0.88	.038	−1.85, 0.98
Psychological Distress (AIMS affect subscale)	−0.24	.377	−1.75, 1.28
Psychological Flexibility (AAQ-II)	−2.56	.073	−6.05, 0.94
Valued Living (BEVS)			
*Health Domain*	0.12	.428	−1.19, 1.42
*Leisure Domain*	−0.29	.718	−1.94, 1.35
*Relationship Domain*	−0.06	.341	−0.35, 0.23
*Work/Community Domain*	−0.59	.250	−2.35, 1.17

Note. *M*_diff_ = mean difference between groups of changes from pre to post; AIMS = Arthritis Impact Measurement Scale; PF = physical functioning; RAPA = Rapid Assessment of Physical Activity; AAQ-II = Acceptance and Action Questionnaire; BEVS = Bulls Eye Values Survey.

## Key Messages Regarding Feasibility

1. Uncertainties existed prior to this study about whether older adults with OA would be interested in participating in a brief behavioral intervention (i.e., Engage-PA) to improve pain, functioning, and increase physical activity, as well as whether the intervention components combining personal values and activity pacing would be acceptable to participants.
2. Engage-PA demonstrated feasibility and acceptability at a priori established benchmarks for continued study, such that 100% accrual was met, attrition was low (5%), and all participants assigned to Engage-PA condition completed all treatment sessions, and 89% of participants reported being “mostly” or “very” satisfied with the program, and secondary outcomes showed promising indicators of arthritis pain and physical functioning improvement as well as increases in physical activity.
3. Given that physical activity can be particularly difficult for older adults, that Engage-PA is feasible and acceptable is notable because all participants reporting having some additional pandemic-specific barriers (e.g., health or finance instability, reduced access and social support for exercising, increased sedentary behavior, reduced physical activity overall) to engaging in physical activity during this time, and the program may be particularly appealing in future effectiveness studies in real-world clinical settings.

## Abbreviations

OA: Osteoarthritis
ACT: Acceptance and Commitment Therapy
PCST: Pain Coping Skills Training
COVID-19: Coronavirus disease of 2019
CIS: COVID-19 Impact Scale
Engage-PA: Engage protocol, physical activity adaptation for OA-related pain
TAU+: Treatment as usual plus fitness tracker
AIMS: The Arthritis Impact Measurement Scale
AAQ-II: The Acceptance and Action Questionnaire-II
CSQ: Client Satisfaction Questionnaire
RAPA: Rapid Assessment of Physical Activity
PARQ-2020: Physical Activity Readiness Questionnaire Plus
REDCap: Research Electronic Data Capture
HIPAA: The Health Insurance Portability and Accountability Act of 1996
CONSORT: Consolidated Standards of Reporting Trials
COPD: chronic obstructive pulmonary disease
M_diff_: Mean difference
CI: Confidence interval
BEVS: Bulls Eye Values Survey
